# Evaluation of genetic differentiation and search for candidate genes for reproductive traits in pigs

**DOI:** 10.5713/ab.23.0297

**Published:** 2024-01-20

**Authors:** Elena Romanets, Siroj Bakoev, Timofey Romanets, Maria Kolosova, Anatoly Kolosov, Faridun Bakoev, Olga Tretiakova, Alexander Usatov, Lyubov Getmantseva

**Affiliations:** 1 Faculty of Biotechnology, Don State Agrarian University, 346493, Rostov region, Oktyabrsky district, Russia; 2 Academy of Biology and Biotechnology named after. DI. Ivanovsky, Southern Federal University, 344090, Rostov region, Rostov-on-Don, Russia

**Keywords:** Breeding, Fst, Genes, Pig, Reproductive Qualities, Single Nucleotide Polymorphism (SNP)

## Abstract

**Objective:**

The use of molecular genetic methods in pig breeding can significantly increase the efficiency of breeding and breeding work. We applied the *F*_st_ (fixsacion index) method, the main focus of the work was on the search for common options related to the number of born piglets and the weight of born piglets, since today the urgent task is to prevent a decrease in the weight of piglets at birth while maintaining high fertility of sows.

**Methods:**

One approach is to scan the genome, followed by an assessment of *F*_st_ and identification of selectively selected regions. We chose Large White sows (n = 237) with the same conditions of keeping and feeding. The data were collected from the sows across three farrowing. For genotyping, we used GeneSeek GGP Porcine HD Genomic Profiler v1, which included 68,516 single nucleotide polymorphisms evenly distributed with an average spacing of 25 kb (Illumina Inc, San Diego, CA, USA).

**Results:**

Based on the results of the Fst analysis, 724 variants representing selection signals for the signs BALWT, BALWT1, NBA, and TNB (weight of piglets born alive, average weight of the 1st piglets born alive, total number born alive, total number born). At the same time, 18 common variants have been identified that are potential markers for both the number of piglets at birth and the weight of piglets at birth, which is extremely important for breeding work to improve reproductive characteristics in sows.

**Conclusion:**

Our work resulted in identification of variants associated with the reproductive characteristics of pigs. Moreover, we identified, variants which are potential markers for both the number of piglets at birth and the weight of piglets at birth, which is extremely important for breeding work to improve reproductive performance in sows.

## INTRODUCTION

Nowadays scientists pay much attention to molecular genetic tools for assessing the breeding and productive qualities of farm animals. The use of certain DNA segments as genetic markers became widespread in the 1980s [1]. Later on, the development of genotyping and sequencing technologies made genome-wide genotyping of single nucleotide polymorphisms (SNPs) more available resulting in an impetus for research genetic basis of complex traits in farm animals as well.

With adopting genomic selection most of the purebred elite lines of farm animals are genotyped using biochips with high and medium density [2]. As a rule, such methods as the genome-wide association study (GWAS) and/or the Selection Signature Analysis [3] are used to evaluate the effects of selection based on the analysis of genomic data. These methods are successfully applied to identify genomic regions in rigorously selected farm animals.

For pigs, it was found that GWAS is a powerful method for identifying genetic variants associated with various breeding traits [4]. However, we should note that identification of reproducible significant associations across the whole genome, GWAS requires significant sampling.

The advantage of studies based on “selection signatures” consists in that they are applicable to relatively small populations under study. One of the “gold standards” for assessing differentiation between the groups is the Fst statistic. Fst is an integral part of descriptive statistics for population estimation, being used in evolutionary biology and clinical genetics, and also to identify genomic loci associated with complex traits [5].

One of the most important factors influencing the economic efficiency of pig breeding is reproduction. The task of identifying loci associated with the reproductive traits of pigs has not lost its relevance over the past decade, but with the advent of new technologies and methods it increasingly attracts researchers. Since genome scanning with further Fst evaluation can identify genome regions subjected to selection we decided to apply the Fst method to identify potential loci associated with reproductive qualities of pigs. The work mainly focused on searching common options related to the fertility of sows and the weight of the litter at birth, since today the urgent task is to prevent a decrease in the weight of piglets at birth with high fertility of sows.

## MATERIALS AND METHODS

### Animals

In accordance with the standard monitoring procedures and recommendations, the specialists of the participating holdings collected tissue samples, following the ethical protocols set out in Directive 2010/63/EU (2010). Ear plucking and handling of pigs were practiced in accordance with the ethical guidelines of the Don state agrarian universitetet, recommendations for compliance with farm and local laws and regulations for the care of pigs (Approval No.: 1-2023-07-11). Collecting ear samples is standard practice in swine production [6].

### Sampling and genotyping

We chose Large White sows (n = 237) with the same conditions of keeping and feeding. The data were collected from the sows across three farrowing. Reproduction indicators on average for 3 farrowing were considered: total number born (TNB), total number born alive (NBA), weight of piglets born alive (BALWT), average weight of the 1st piglets born alive (BALWT1). Data processing was performed with the R studio program, in filtering the data outliers greater than 3 sigma were removed. For each trait, 2 groups were formed: with low productivity, below the 25% quantile; and high productivity, above the 75% quantile.

For genotyping, we used GeneSeek GGP Porcine HD Genomic Profiler v1, which included 68,516 SNPs evenly distributed with an average spacing of 25 kb (Illumina Inc, San Diego, CA, USA). The total genotyping rate was 0.99. Genomic data was filtered using the Plink 1.9 [7] in accordance with the following parameters --geno 0.1 --mind 0.1 --maf 0.05 --hwe 1e-3 --indep-pairwise 50 5 0.8.

### Data analysis

To identify potential loci for the reproductive qualities of pigs, we used the *F*_st_ method implemented in Plink 1.9 [7]. This method identifies differences in allele frequencies between groups with low and high productivity for each trait and records *F*_st_ estimates for each autosomal diploid variant (calculated using the method presented by Cockerham and Weir [8]. The *F*_st_ values, corresponding to 0.99%, were identified and translated into genomic positions of Sus scrofa 11.1, and the content of each region was analyzed. The search of quantitative trait locus (QTL) performed in PigQTLdb and also a literature search was also carried out manually for the presence of data on the associations of genes with any traits in humans and animals.

## RESULTS

The traits characterizing the fertility of sows and the weight of piglets at birth were of certain interest. For this research we divided the indicators for all features into low, medium and high ones, taking into account the quantiles of 0% to 25%, 25% to 75%, and 75% to 100%, respectively. In searching selection signatures we used only extreme phenotypes of L and H groups. The average values for all sows in the studied sample, and as well as in the groups with low and high productivity are presented in Table 1.

**Figure 1:**
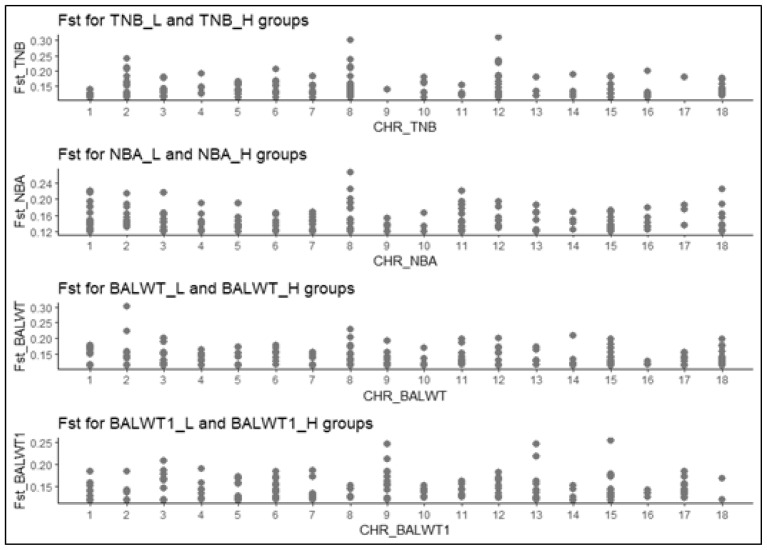
Emissions based on the results of the *F*_st_ analysis between groups with high and low indicators for the TNB, NBA, BALWT, and BALWT1 phenotypes. H, group with the highest values for the studied phenotype; L, group with minimal indicators for the studied phenotype; CHR, chromosome; *F*_st_, fixation index (an indicator of differentiation of populations due to their genetic structure); TNB, total number born; NBA, total number born alive; BALWT, weight of piglets born alive; BALWT1, average weight of the 1st piglets born alive.

Based on the results of the *F*_st_ analysis we identified 724 outliers exceeding 0.99%. These variants represent selection signals for the BALWT, BALWT1, NBA, and TNB traits. In general, outliers are presented on all chromosomes; this is especially clearly seen for BALWT1. For other traits we can identify individual chromosomes with the largest number of outliers. Thus for BALWT and TNB the largest number of outliers is located in SSC8, for NBA – in Sus scrofa chromosomes 3 (SSC3) (Figure 1). The outliers are represented by various variants of nucleotide substitutions, to a greater extent these are intron and intergenic variants, but there are also upstream gene, non coding transcript exon, downstream gene and 3 prime UTR variants (Supplementary file).

For BALWT1 and BALWT, 197 and 201 variants, were identified respectively, of which 22 SNPs are common for both traits. For TNB and NBA (219 and 198) variants were found respectively, while 46 SNPs were common for these traits. Between the fertility traits (NBA and TNB) and piglet weights (BALWT1 and BALWT), common variants were also established (Table 2).

Two variants turned out to be common between BALWT1 and NBA: rs81379527 (SSC3: 27441840), an intron variant of the xylosyltransferase 1 (*XYLT1*), and rs80934876 (SSC4: 354634), an intron variant of the tonsoku like, DNA repair protein (*TONSL*). *XYLT1* is expressed in chondrocytes during embryonic development and encodes xylosyltransferase 1, with its functions associated with the synthesis of proteoglycans. In the research of Bergfelder-Drüing et al [9] an association between the *XYLT1* gene (rs81379421) and number piglets born alive was revealed. In addition, *XYLT1* is a potential candidate gene for the short stature and dwarfism syndrome. *TONSL* variants have deleterious effects at multiple stages of embryonic and postnatal development.

There are four variants located in SSC8, turned out to be common between BALWT1 and TNB, of which rs321611489 and rs81404839 are intron variants of the potassium voltage-gated channel interacting protein 4 (*KCNIP4*) gene. The effect of *KCNIP4* gene variants on porcine NBA was reported by He et al [10].

There are three common variants have been identified between BALWT and NBA, intron variants rs327523214 (SSC3: 36974365) of the RNA binding *FOX-1* gene; intergenic variants rs80838609 (SSC14: 139576563); synonymous variant of the thromboxane A synthase 1 (*TBXAS1*) rs81248107 gene (SSC18: 9717477). The *TBXAS1* enzyme is involved in several pathophysiological processes, including hemostasis, cardiovascular disease, and apoplexy. In addition, it is assumed that the enzyme is involved in the regulation of uterine and intrauterine blood flow.

Two variants common for BALWT and TNB have been identified: intron variant rs81256424 (SSC8: 65110831) of the ENSSSCG00000063524 gene and intron variant rs81330142 (SSC11: 18043223) of the emopamil binding protein-like (*EBPL*) gene. The relationship of the *EBPL* gene with the body weight of young bulls was highlighted in the work of Lindholm-Perry [11].

Besides 5 SNPs were noted for more than two traits (Table 2). Thus, intergenic variant rs334075913 showed a selection signal for BALWT1, NBA, and TNB. this variant is localized in SSC2: 138901377. QTL#106221 associated with piglet mortality was previously defined in this area. In close proximity to this variant, the secreted protein acidic and cysteine rich (*SPARC*) gene (*SPOCK1* (osteonectin), SSC2: 139018177 – 139523601) is localized, which was identified as a new candidate gene for the Menarche age and is associated with the onset of female reproductive life, cattle, humans, and sheep fecundity [12] and ovulation rate in pigs [13].

Intergenic variant rs81476874 showed a selection signal for BALWT1, BALWT, and TNB traits. This variant is localized in SSC8:12351405. This area intersects with QTL #24282 associated with litter size and QTL #645 affecting the plasma follicle-stimulating hormone (*FSH*) and ovulation rate in females [14]. Intergenic variant rs328047631 (SSC8: 13883790) for BALWT1, BALWT, and NBA and intergenic variant rs81401114 (SSC8: 11570387) for BALWT1, NBA, and TNB were also found in the eighth chromosome. This option intersects with a number of QTLs, among which it is interesting to note QTL#24282 for Litter size; QTL#325 [15] and QTL:656 for Average daily gain; QTL#653, QTL#654, QTL#655, QTL #21254 and QTL#21253 [16] for Body weight. Besides, the transmembrane anterior posterior transformation 1 (*TAPT1*), (SSC8: 11365423-11415699) and lim domain binding 2 (*LDB2*), (SSC8: 11641060..12037388) genes are localized in close proximity to the rs81401114 variant (SSC8: 11570387). *TAPT1* encodes the evolutionary conservative transmembrane protein anterior posterior of transformation 1. The studies [17] show the involvement of *TAPT1* in the basis of a complex congenital syndrome clinically manifested by lethal skeletal dysplasias and ciliopathy. This syndrome is characterized by fetal death and multiple congenital malformations.

The *LDB2* gene plays a regulatory role in retinal development and the cell cycle, but its biological role remains unclear. The association of the *LDB2* gene is characterized by the body weight of chickens (of commercial broiler chickens) and birth weight in Cashmere goats [18].

Synonymous variant rs81211492 showed a selection signal for BALWT, NBA, and TNB. This variant is localized in the aminopeptidase puromycin sensitive (*NPEPPS*), (SSC12: 23804839..23892055) gene. This gene encodes the puromycin-sensitive aminopeptidase playing the neuroprotective role [19]. Besides, the protein is involved in the regulation of the cell cycle in mammals, and it is required for meiosis exit and anteroposterior polarity in single-celled Caenorhabditis elegans embryos and mice deficient in puromycin-sensitive aminopeptidase are smaller and have reproductive problems [20]. According to the QTLdb database, rs81211492 overlaps with 42 QTLs associated with various functions, but 5 QTLs (QTL:5261, QTL:5227 [21], QTL:4254 [22], QTL:6479, QTL:6472 [23]) are responsible for tea number.

Moreover, the intron variant rs80883327 (SSC17: 1204066) of the deleted in liver cancer 1 (*DLC1*) gene showed a selection signal for all four traits. *DLC1* is considered a tumor suppressor gene in various human cancers. The functional features of *DLC1* in pigs are not studied enough. According to an open-access pig expression map [24], the *DLC1* expression is enhanced in retina, fallopian tube, testis and upper respiratory system. In other species, evidence of the role of *DLC1* in embryogenesis has been obtained. The *DLC1* gene plays a critical role in the regulation of cellular functions during the early development of mice. According to Durkin et al [25] homozygous mica with *DLC1* knockout died around 10.5 days of embryonic development. The role of *DLC1* in spermatogenesis and male fertility in mice is presented in Okitsu [26]. The *DLC1* protein is also thought to play an important role in the development of the placenta [27].

## DISCUSSION

Reproductive traits play a leading role in the economics of pig production. In this regard, the interest in the biology of these traits has not waned for decades. The main indicators of the reproductive performance of sows are the total number of piglets at birth (TNB) and the number of live piglets at birth (NBA), since these signs are measured ones and there are no particular difficulties in taking them into account. However, these traits have a very low inheritance rate and an extremely complex biology. In fact, these signs combine all the processes associated with the reproductive cycle of sows (ovulation, fertilization, implantation, prenatal survival, uterine capacity, etc.).

In addition, along with the fertility of sows the matter of piglet weight at birth is arising. On the one hand, the increase in the number of piglets at birth is believed to be directly related to the decrease in the weight of piglets. On the other hand, there appears more evidence that the decrease in the weight of piglets at birth does not depend directly on their number, but more related to the capacity of the uterus and the body of the uterus, the ability to provide energy costs for the full development of offsprings in the embryonic period.

Since these traits have low heritability, their genetic architecture is rather interesting. The growth of GWAS research on the genetic architecture of reproduction was observed at the end of 2017 after the publication of an updated version of the pig genome [10]. A large amount of data obtained today on the basis of GWAS showed a low reproducibility of the results, which can be explained by the individual characteristics of the genetic structure of populations (allele frequencies, linkage disequilibrium), but also by the need for a large sample of animals, this is especially important for traits with low heritability.

In our work to assess the genetic architecture of sow reproduction we decided on four main traits TNB, NBA, BALWT1, and BALWT, and chose the selection signature search approach based on the *F*_st_ method as a method for identifying genetic variants. As a result, we found 724 variants for all features. Selection signals for two traits was shown in 79 variants. To a greater extent the variants overlapped between TNB and NBA, which is quite expected since the biology of these traits has so much in common. Generic variants between BALWT1 and BALWT require more effective analysis in the future, as they may provide some clarity on the relationship between litter weight and average birth weight per piglet. Besides, a number of variants responsible for both the mass of the nest and the number of piglets were identified. These variants are of interest as potential markers for assessing correlations between the number of piglets and their weight. Most of them are localized in genes (but nearby genes are also considered in the case of intergenic variants). The functional characteristics of genes and their associative relationships with productivity traits presented in the literature indicate their connection, to a certain degree with the reproductive process, either in pigs, or the data are presented for other species. The functions of genes are associated with the embryonic process, the survival of embryos, as well as various pathologies, further associated with a growth decrease. All selection variants directly related to the number and weight of piglets intersect with different QTLs, including for average daily gain and body weight. Some of these genes were previously directly noted in associative studies with sow fertility and are reviewed by Bakoev et al [28].

## CONCLUSION

Thus, our work resulted in identification of variants associated with the reproductive characteristics of pigs. Moreover, we identified variants which are potential markers for both the number of piglets at birth and the weight of piglets at birth, which is extremely important for breeding work to improve reproductive performance in sows.

## Figures and Tables

**Table 1:** Average values for all sows in the study sample, as well as in groups with low and high productivity

Trait	Group^1)^	Mean	Se	Sd	Min	Max
TNB	All	13.39	0.118	1.814	8.00	18.50
	H	15.69	0.113	0.874	14.67	18.50
	L	11.36	0.112	0.966	8.00	12.33
NBA	All	12.55	0.122	1.882	7.00	17.67
	H	14.60	0.107	0.928	13.67	17.67
	L	10.25	0.121	0.994	7.00	11.33
BALWT	All	14.82	0.155	2.385	9.15	20.53
	H	17.87	0.130	1.006	16.37	20.53
	L	11.78	0.144	1.126	9.15	13.25
BALWT1	All	1.19	0.011	0.173	0.70	1.56
	H	1.42	0.008	0.061	1.33	1.56
	L	0.98	0.011	0.083	0.70	1.07

Se, standard error; Sd, standard deviation; TNB, total number born; NBA, total number born alive; BALWT, weight of piglets born alive; BALWT1, average weight of the 1st piglets born alive.

1) All, entire group being studied; H, group with the highest values for the studied phenotype; L, group with minimal indicators for the studied phenotype.

**Table 2:** General single nucleotide polymorphism between piglet weight (BALWT1 and BALWT) and fertility traits (TNB and NBA)

CHR	Position	*F*_st_ BALWT1	*F*_st_ BALWT	*F*_st_ TNB	*F*_st_ NBA	Gene
2	138901377	0.139	-	0.112	0.147	*SPARC*
3	27441840	0.120	-	-	0.119	*XYLT1*
3	36974365	-	0.201	-	0.151	*RNA binding fox-1*
4	354634	0.120	-	-	0.124	*TONSL*
8	16314430	0.128	-	0.150	-	*KCNIP4*
8	16332926	0.142	-	0.125	-	*KCNIP4*
8	16568164	0.123	-	0.125	-	*ADGRA3*
8	17543929	0.123	-	0.125	-	*PPARGC1A*
8	65110831	-	0.120	0.149	-	*ENSSSCG00000063524*
8	11570387	-	0.132	0.209	0.139	*TAPT1, LDB2*
8	12351405	0.128	0.230	0.150	-	*LDB2, QDPR*
8	13883790	0.128	0.180	-	0.125	*-*
11	18043223	-	0.123	0.118	-	*EBPL*
12	23890636	-	0.112	0.231	0.128	*NPEPPS*
14	139576563	-	0.115	-	0.124	*TCERG1L*
17	1204066	0.142	0.155	0.178	0.185	*DLC1*
18	9717477	-	0.123	-	0.125	*TBXAS1*

*F_st_*, fixation index (an indicator of differentiation of populations due to their genetic structure); BALWT1, average weight of the 1st piglets born alive; BALWT, weight of piglets born alive; TNB, total number born; NBA, total number born alive.
