# Supplementary material for: Evaluation of genetic differentiation and search for candidate genes for reproductive traits in pigs
Source: Anim Biosci. 2024 Jan 20;37(5):832–8. doi: 10.5713/ab.23.0297 (PMC11065708; doi:10.5713/ab.23.0297)
Supplement: Supplementary file 1 [file ab-23-0297-Supplementary-1.pdf]

CHR	POS	Fst	variants (of nucleotide substitutions)
1	13912672	0.150684932	intron_variant
1	78788434	0.12	intergenic_variant
1	81033340	0.138846154	intergenic_variant
1	94198691	0.183098592	intergenic_variant
1	94816562	0.127877238	intergenic_variant
1	100286992	0.12	intron_variant
1	202129950	0.117718215	intron_variant
1	202242191	0.158024691	intron_variant
1	258899089	0.12	non_coding_transcript_exon_variant
2	14074164	0.12	intergenic_variant
2	21691721	0.183098592	intergenic_variant
2	52753366	0.135135135	intergenic_variant
2	125910374	0.117718215	intron_variant
2	128925149	0.141065831	intergenic_variant
2	138901377	0.138846154	intergenic_variant
3	27441840	0.12	intron_variant
3	92615218	0.178181818	intergenic_variant
3	93170397	0.166666667	intron_variant
3	94034553	0.207792208	downstream_gene_variant
3	94340673	0.185354691	intergenic_variant
3	94771351	0.162422037	intron_variant
3	95160980	0.163879599	non_coding_transcript_exon_variant
3	95577841	0.145454545	intergenic_variant
3	99815164	0.166666667	intergenic_variant
3	114349621	0.117718215	intergenic_variant
3	125980682	0.163285024	intergenic_variant
4	354634	0.12	intron_variant
4	3672482	0.189597459	intergenic_variant
4	3844265	0.158024691	intergenic_variant
4	3987272	0.134127789	intergenic_variant
4	4564148	0.142378344	intron_variant
4	4928177	0.141065831	intergenic_variant
4	5253019	0.122979714	intergenic_variant
4	5395316	0.122979714	intergenic_variant
4	21146108	0.12	intergenic_variant
4	128019716	0.158024691	intergenic_variant
5	5570166	0.12	intron_variant
5	5634814	0.127877238	intron_variant
5	5672567	0.172058301	intron_variant
5	8208348	0.158024691	intron_variant
5	8812727	0.158024691	intergenic_variant
5	10589336	0.117718215	3_prime_UTR_variant
5	10597987	0.12	upstream_gene_variant
5	11435745	0.117718215	intron_variant
5	12544917	0.12	intron_variant
5	12750388	0.166666667	downstream_gene_variant
5	16650384	0.127877238	intron_variant
5	66474878	0.154731458	intron_variant
5	67662549	0.122979714	intron_variant

5	89256566	0.12	intergenic_variant
5	99093875	0.127623369	intron_variant
6	8017269	0.12	downstream_gene_variant
6	8160571	0.172058301	intergenic_variant
6	19302391	0.125	3_prime_UTR_variant
6	25884885	0.155145362	intron_variant
6	25911255	0.143879854	intron_variant
6	30220425	0.183098592	intergenic_variant
6	35240005	0.138846154	intron_variant
6	67959109	0.135135135	intron_variant
6	68032093	0.172058301	intron_variant
6	69845510	0.150684932	intergenic_variant
6	146250600	0.166666667	intron_variant
6	146425347	0.12	intron_variant
6	151474038	0.127877238	intergenic_variant
6	153777353	0.127877238	intron_variant
6	154252493	0.123152709	non_coding_transcript_exon_variant
6	154865983	0.143936926	downstream_gene_variant
6	154963324	0.127368421	intron_variant
6	154970954	0.127368421	intron_variant
7	3422988	0.130806941	intron_variant
7	4867925	0.127623369	intron_variant
7	4930409	0.122979714	intron_variant
7	5507216	0.185354691	intergenic_variant
7	6703380	0.170491803	intergenic_variant
7	15568984	0.127623369	intron_variant
7	19833122	0.127623369	intron_variant
7	30234691	0.127368421	intron_variant
7	98565888	0.125922282	intergenic_variant
7	99716433	0.132352941	downstream_gene_variant
7	109877373	0.130806941	intergenic_variant
7	115310787	0.12	intron_variant
7	117737064	0.125922282	intron_variant
8	11290812	0.145454545	intron_variant
8	12279651	0.127623369	intergenic_variant
8	12351405	0.127623369	intergenic_variant
8	13047394	0.125	intergenic_variant
8	13883790	0.127623369	intergenic_variant
8	16314430	0.127623369	intron_variant
8	16332926	0.142472394	intron_variant
8	16568164	0.122979714	intergenic_variant
8	17543929	0.122979714	intergenic_variant
8	81039805	0.127623369	intergenic_variant
8	81474626	0.125922282	intergenic_variant
8	120600278	0.150684932	downstream_gene_variant
8	123151295	0.125	intergenic_variant
8	124972762	0.127877238	intergenic_variant
8	130360804	0.123152709	intron_variant
9	3377757	0.12	upstream_gene_variant
9	24006973	0.142196943	intergenic_variant

9	46839557	0.123152709	intron_variant
9	66463552	0.155145362	upstream_gene_variant
9	126707269	0.154731458	intergenic_variant
9	127054823	0.161290323	intron_variant
9	127505735	0.184184644	intron_variant
9	127939523	0.181735911	intron_variant
9	128047578	0.211945565	intron_variant
9	128431815	0.154731458	3_prime_UTR_variant
9	132436894	0.16154045	intron_variant
9	132612394	0.246153846	intron_variant
9	132821695	0.170491803	intron_variant
9	133948499	0.172058301	intergenic_variant
9	136517022	0.150684932	intron_variant
10	5106897	0.150684932	intergenic_variant
10	20635267	0.135135135	intron_variant
10	22977911	0.123152709	intergenic_variant
10	23853572	0.141065831	intron_variant
10	23996663	0.127877238	intron_variant
10	24927504	0.150684932	intron_variant
10	40499841	0.14619883	intergenic_variant
10	46538968	0.135135135	intron_variant
10	58174508	0.141065831	intergenic_variant
10	65725566	0.138846154	intergenic_variant
11	13585863	0.155145362	intergenic_variant
11	19717338	0.142196943	intergenic_variant
11	24408433	0.130806941	intergenic_variant
11	24781270	0.142378344	downstream_gene_variant
11	75670479	0.16064379	intergenic_variant
11	75750970	0.127368421	intron_variant
11	75806087	0.125	intron_variant
12	9511551	0.127877238	intergenic_variant
12	10162364	0.150684932	intergenic_variant
12	13279137	0.163879599	intergenic_variant
12	13351767	0.162422037	intergenic_variant
12	14993375	0.14619883	downstream_gene_variant
12	18834021	0.134127789	intron_variant
12	18975689	0.145454545	downstream_gene_variant
12	19502943	0.125	downstream_gene_variant
12	47703165	0.181735911	intron_variant
12	48780296	0.164672365	upstream_gene_variant
12	48874241	0.134127789	intron_variant
12	57456741	0.125	intergenic_variant
12	57630692	0.169761273	3_prime_UTR_variant
12	57739994	0.163285024	intergenic_variant
12	57829316	0.127623369	intergenic_variant
12	57950275	0.14619883	non_coding_transcript_exon_variant
12	58026707	0.123943662	intron_variant
13	1417981	0.155145362	intron_variant
13	6548446	0.122979714	intergenic_variant
13	6718277	0.12	intergenic_variant

13	8659932	0.16064379	non_coding_transcript_exon_variant
13	9093001	0.141065831	intergenic_variant
13	9111717	0.246153846	intergenic_variant
13	9158302	0.217391304	upstream_gene_variant
13	197273280	0.135135135	intron_variant
13	198895921	0.125	intergenic_variant
13	200633685	0.125922282	intergenic_variant
13	202901702	0.125	downstream_gene_variant
14	17358204	0.150684932	intron_variant
14	19508478	0.12	intergenic_variant
14	122803682	0.117718215	intron_variant
14	130464426	0.125922282	intergenic_variant
14	136408215	0.142378344	intron_variant
15	1091163	0.178181818	intergenic_variant
15	1177587	0.253731343	upstream_gene_variant
15	1770319	0.123943662	downstream_gene_variant
15	10722037	0.125	intron_variant
15	11245685	0.175897748	intron_variant
15	17343229	0.130806941	intron_variant
15	29693692	0.125	intron_variant
15	34728389	0.135135135	intron_variant
15	102237263	0.117718215	upstream_gene_variant
15	122374213	0.123943662	intergenic_variant
15	123040633	0.143936926	intergenic_variant
15	123132577	0.170491803	intergenic_variant
15	134307672	0.143879854	downstream_gene_variant
15	138319532	0.142196943	intergenic_variant
15	138424165	0.127623369	intron_variant
16	1990610	0.134127789	intergenic_variant
16	5408721	0.132352941	intron_variant
16	5537820	0.142196943	intergenic_variant
16	22790742	0.135135135	intron_variant
16	62326007	0.125922282	intergenic_variant
16	64771399	0.125	upstream_gene_variant
17	1204066	0.142378344	intron_variant
17	5029462	0.130806941	intron_variant
17	5341124	0.151702786	intron_variant
17	5422611	0.125	upstream_gene_variant
17	10072333	0.150684932	intron_variant
17	14317920	0.123152709	intergenic_variant
17	16661414	0.143879854	intergenic_variant
17	17003637	0.135135135	upstream_gene_variant
17	19474175	0.138846154	intron_variant,non_coding_transcript_variant
17	32012959	0.155145362	intron_variant
17	32836412	0.183098592	downstream_gene_variant
17	48186419	0.172058301	intron_variant
18	47495588	0.12	downstream_gene_variant
18	51579645	0.166666667	intron_variant
